# Reduced Carbon Emissions and Chain Elongation during Mixotrophic Fermentation of a Biomass Feedstock

**DOI:** 10.1021/acsomega.6c06316

**Published:** 2026-10-08

**Authors:** Myra X. Afzal, Maria L. Bonatelli, Sabine Kleinsteuber, Heike Sträuber, Flávio C. F. Baleeiro

**Affiliations:** † Department of Microbial Biotechnology, 28342 Helmholtz Centre for Environmental Research − UFZ, Permoserstr. 15, 04318 Leipzig, Germany; ‡ Thayer School of Engineering, 145792 Dartmouth College, 15 Thayer Drive, Hanover, New Hampshire 03755, United States; § Department of Engineering and Natural Sciences, Merseburg University of Applied Sciences, Eberhard-Leibnitz-Str. 2, 06217 Merseburg, Germany; ∥ Institute of Biology, Department of General Genetics, 99019 Martin Luther University Halle-Wittenberg, Weinbergweg 10, 06120 Halle (Saale), Germany

## Abstract

Anaerobic fermentation of biomass feedstocks using open cultures is a promising technology to produce platform carboxylates. Syngas, a mixture of H_2_, CO_2_, and CO, can be sourced sustainably and used to supplement biomass feedstocks as a source of acetyl-CoA, an intermediate for carboxylate chain elongation. To test this, syngas and corn silage were provided to a 10-L semicontinuous fermenter for 208 days of operation in a first-of-a-kind study at this scale. After acclimation to syngas, a 2-fold reduction in average CO_2_ production rate (0.097 vs 0.21 g L^–1^ d^–1^) was observed over a period of 42 days in comparison to a control. Syngas cofeeding also increased average production rates of *n*-butyrate (C4) and *n*-caproate (C6) by 74 and 27%, respectively, although these effects were observed at relatively low C6 concentrations up to 4 g L^–1^. Abundances of *Megasphaera* and *Dialister* showed significant correlation (*p* < 0.05) to consumption of H_2_, CO_2_, and CO as well as production of C3, C4, C5, or C7, suggesting involvement of these genera in mixotrophic metabolism. A feasibility analysis showed that syngas recirculation could return additional 1.54 USD m_broth_^–3^ while costing 1.26 USD m_broth_^–3^ and avoiding 1.81 kg CO_2 eq._ m_broth_^–3^ in emissions compared to heterotrophic fermentation. With still much room to increase syngas consumption and carboxylate production, we propose mixotrophic fermentation as a “low-tech” technology to turn fermenters into decentralized industrial carbon sinks.

## Graphical abstract

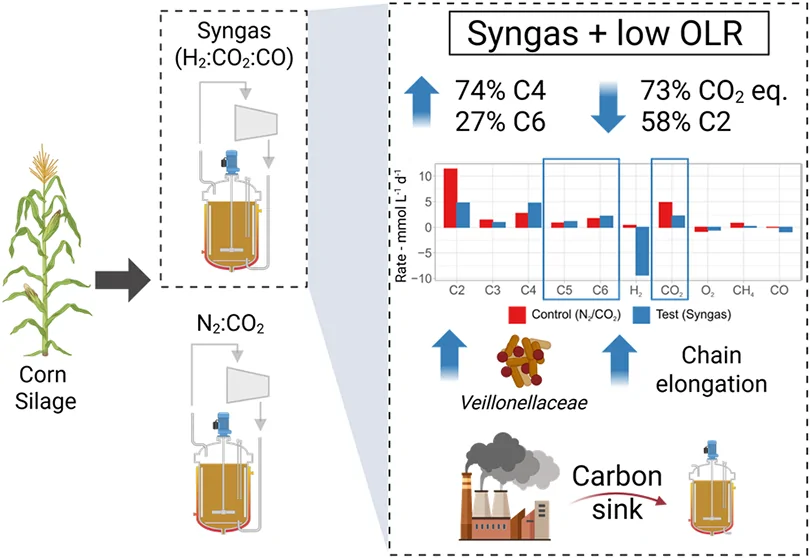

## Introduction

Anaerobic fermentation of abundant organic feedstocks, such as lignocellulosic biomass, offers a promising platform for the production of value-added chemicals. One such case is the conversion of agricultural residues to useful, high-value products such as medium-chain carboxylates (MCCs) by anaerobic microbial communities capable of chain elongation (CE) metabolism. MCCs are monocarboxylates with linear or branched (iso) chains containing six to 12 carbon atoms in the backbone.^1,2^ MCCs have a vast spectrum of applications in industries (e.g., cosmetics, food and feed ingredients, pharmaceuticals, and agrochemicals) and are currently produced from coconut oil, palm kernel oil, or fossil oil.^3,4^ Compared to other MCCs, *n*-caproate (C6) and *n*-caprylate (C8) are of particular interest because of the relatively high quantity produced by anaerobic microbial communities and the potential to be extracted by simpler and cheaper downstream processing in comparison to their short-chain counterparts.^5^ Using open cultures for anaerobic fermentation reduces sterility requirements during production compared with defined cultures. It also provides a way to use low-cost and complex feedstocks.

Despite these benefits, there are several challenges to overcome for the fermentation of lignocellulosic biomass to become a competitive chemical production route. Lignocellulosic feedstocks, such as corn silage, generally present a relative scarcity of electron donors (EDs; e.g., ethanol and lactate) in comparison to electron acceptors (EAs; e.g., acetate and *n*-butyrate). This scarcity of ED decreases selectivity of longer-chain, more desired chemical products,^6–9^ such as caproic acid, and favors competing metabolic pathways.^5,10^ Variable availability of nearby feedstock also limits biorefineries from profiting from economies of scale.^11^ To address the challenges of ED scarcity and variable substrate availability, syngas, a mixture of hydrogen (H_2_), carbon dioxide (CO_2_), and carbon monoxide (CO), has been proposed as a cosubstrate to microbial communities producing MCCs.^5,12^ Microbial communities growing autotrophically on syngas or a combination of its components without organic feedstock are typically limited to production of ethanol or short-chain carboxylates due to thermodynamic and kinetic bottlenecks.^13^ When syngas is cofed with a complex organic feedstock, it is possible to simultaneously enrich heterotrophic and autotrophic metabolic functions in a microbial community, profiting from advantages of both worlds and leading to a *de facto* mixotrophic fermentation.

The addition of external H_2_ alone can steer selectivity into more reduced products,^14,15^ and this gas has been previously shown to be a promising ED supplement for CE when cofed with corn silage in batch cultures,^16^ although the mechanism through which H_2_ can improve MCC production remains elusive. In terms of caproate production, *Megasphaera elsdenii* has long been known as a C6-producer that has the capability to utilize H_2_,^17^ and the genus *Megasphaera* has been shown to be a predominant community member in systems enriched with H_2_/CO_2_ ^16^ and CO^18^ during fermentation of complex biomass feedstocks, making it an interesting candidate for CE in mixotrophic fermentation. In addition, H_2_ offers a bridge to couple MCC production with the renewable power grid, be it via ex situ water electrolysis or with bioelectrochemical systems.^19^ However, the use of H_2_ alone does not take advantage of beneficial components found in syngas such as CO, which can also act as an ED. CO and impurities found in certain industrial syngas streams (e.g., ethylene, C_2_H_4_; and acetylene, C_2_H_2_)^20^ also act as methanogenesis inhibitors. We have previously observed that the addition of CO or small doses of C_2_H_4_ inhibits methanogenesis,^16^ and when both gases were combined, a complementary effect was observed. This is a relevant strategy for the stable, long-term, economically viable suppression of methanogens, in particular as hydrogenotrophic methanogens are found in systems with abundant, exogenous H_2_.^10^ Although several other methanogenesis inhibitors are known, these molecules are either expensive or hard to recycle,^16^ and inoculum pretreatment does not work with fermentation of unsterile feedstocks. Organisms that realize the Wood-Ljungdahl pathway produce acetyl-CoA from syngas, which can be used intracellularly as an educt for CE or converted to ethanol and exported to chain elongators, further emphasizing the potential of using syngas in CE.^21^ Previously, syngas has been shown to be an effective ED source when cofed with organic model feedstocks such as lactate and acetate in mineral media.^22^ We expand upon this work by demonstrating the efficacy of syngas as an effective ED source and methanogenesis inhibitor in a system using an industrially relevant lignocellulosic biomass.

Carbon fixation is another promising aspect of mixotrophic fermentation. This can be done in two ways: by steering the fermentation away from CH_4_ and CO_2_ production by inhibiting methanogenesis and promoting the utilization of CO_2_. These approaches not only reroute electrons toward CE but also have the potential to reduce carbon emissions of the whole process. The mixotrophic and autotrophic members of a microbial community are able to utilize the CO_2_ and H_2_ produced by heterotrophic fermentation in addition to consuming any externally supplied syngas. This syngas-consuming capability has been shown in batch cultures in several studies.^14,16,23–25^ The ability to steer a mixotrophic community toward syngas and synthetic growth medium consumption in a stirred-tank reactor setting has also been shown.^22^ Even the external supply of CO_2_ without the other common syngas components is able to enrich some carbon-fixing functions as shown by de Leeuw et al.^26^ and Roghair et al.^27^

The supply of syngas or a combination of its components also has implications for solventogenesis (i.e., the formation of alcohols) as part of anaerobic fermentation. Certain acetogens and chain-elongating microbes can perform solventogenesis by reducing carboxylic acids produced in CE to their corresponding alcohols using H_2_ or CO as electron donors.^28–30^ Solventogenesis has been observed to be induced and tunable by parameters such as pH,^30–32^ temperature,^33^ and CO_2_ partial pressure.^26,27^ The potential for tunable alcohol production following CE makes solventogenesis an interesting avenue to explore in mixotrophic fermentation.

Despite the growing body of the literature in the field of mixotrophic fermentation, no studies have yet shown the long-term feasibility of this technology in open systems using real, complex organic feedstocks and complete syngas mixtures with H_2_, CO_2_, and CO.

In this study, we present and evaluate the feasibility of mixotrophic fermentation with syngas and lignocellulosic biomass (corn silage, as a feedstock model) to produce MCCs in a 10-L semicontinuous fermenter harvested and fed twice per week. Working at this scale not only tests process scalability but also enables us to move beyond batch-scale studies with synthetic media and instead operate a long-term, continuous reactor with complex lignocellulosic feedstock. To our knowledge, this type of study has not yet been conducted with mixotrophic fermentation. Gas composition, temperature, pH, and organic loading rate (OLR) are discussed with regard to their effects on product yield, gas consumption, carbon fixation, and MCC concentration. In addition to discussing process parameters for CE in mixotrophic fermentation and the potential of this technology as a carbon-fixing process, we also discuss the roles of key microbes, such as the genera *Megasphaera* and *Dialister*, in mixotrophic fermentation. The results of this study have the potential to lay groundwork toward scale-up and application of this sustainable technology.

## Materials and Methods

### Bioreactor Operation

Two identical 15-L anaerobic stirred-tank bioreactors used previously^34^ and retrofitted with gas recirculation systems were operated for 208 days. Reactors were stirred at 150 rpm and operated at a 10-L working volume, a hydraulic retention time of 10 days, and feeding every 3.5 days. Each reactor was equipped to continuously recirculate 80:20 v/v N_2_:CO_2_ (control reactor), 80:20 H_2_:CO_2_ (test reactor), or approximately 75:18:7 H_2_:CO_2_:CO (test reactor) from an 18-L flexible gas reservoir. Gas was delivered to the broth via aquarium spargers at the bottom of the reactor at a rate of approximately 1.5 L min^–1^ using a diaphragm pump (model NMP830 from KNF Neuberger GmbH, Germany) adapted with a housing for physical and electrical protection. The reactor configuration was similar to the continuous reactor described by Baleeiro et al.^35^ To ensure consistent recirculation of desired gases and to prevent overpressurization due to microbial gas production, gas reservoirs were emptied using a vacuum pump and refilled every 7 days with the approximate compositions described above. The partial pressures of noninert gases across the experiment are shown in Figure S1. The gas balance in the first 70 days was not used to show gas accumulation data because the He concentrations were sometimes too low and the data were not reliable. The presence of N_2_ was used as an indirect measurement of O_2_ contamination from air. This method to estimate O_2_ contamination has been described in a similar system before.^36^ For detailed information on reactor operation, please refer to the Supporting Information (SI).

Reactors were operated at 32 °C and pH 5.5, with the exception of days 146–181, when pH and temperature alternated between 32 °C/pH 5.5 and 28 °C/pH 4.8 every 3.5 or 7 days. A detailed description of reactor operation phases and process conditions can be found in Table S1. OLR was maintained at either 3.6 or 7.2 g volatile solids (VS) L^–1^ d^–1^ with a constant hydraulic retention time of 10 d. These two OLR values were named “low” and “high”, respectively. Corn silage from Neichen, Germany, was used as substrate, and sludge from a mesophilic biogas reactor fed with corn silage and cow manure operated by the *Deutsches Biomasseforschungszentrum - DBFZ* (Leipzig, Germany) was used as inoculum. The inoculum and the corn silage were sampled in triplicate to determine total solids (TS), VS, ammonia nitrogen content (NH_4_–N), and pH and are reported in Table S2. Microbial composition of the inocula used for start-up and on day 104 is presented in Figure S2.

### Sample Collection and Chemical Analysis

Gas composition (He, H_2_, CO_2_, C_2_H_4_, CH_4_, O_2_, N_2_, and CO) was monitored five times per week using two gas chromatography (GC) systems (MicroGC Fusion, Inficon, Germany; PerkinElmer). Analysis by PerkinElmer GC was done as described by Logroño et al.^37^ Analysis by MicroGC was performed as described by Mohr et al.,^38^ where the sampling volume was 2 mL. Broth was sampled from the reactor harvest prior to feeding, which occurred twice a week. Broth was analyzed for pH, TS, VS, NH_4_–N, and organic acids and alcohols content. TS and VS analyses were done as described by Strach.^39^ NH_4_–N content was measured as described by Strach.^40^

The chemical content of the broth was measured by high-performance liquid chromatography (HPLC) and by a GC system coupled to a flame ionization detector with samples preceding a derivatization by esterification (ester-GC) adapting methods from Apelt^41^ and Apelt,^42^ respectively. Modifications to these methods are described in the SI. Measured compounds include formate (C1), acetate (C2), lactate (LAC), ethanol (EtOH), propionate (C3), *n*-propanol (PropOH), *n*-butyrate (C4), *i*-butyrate (iC4), *n*-butanol (ButOH), *n*-valerate (C5), *i*-valerate (iC5), *n*-pentanol (PentOH), *n*-hexanoate (C6), *i*-hexanoate (iC6), *n*-heptanoate (C7), and *n*-caprylate (C8).

Rates in this study are average rates of production or consumption specific to the broth volume (10 L). Average rates calculated in mmol of electron equivalents (e^–^ mmol) were used to compare chemicals in the gas and liquid phases. The conversion factors for realizing molar, electron, carbon and material balances are presented in Table S3. The original experimental data and calculated rates are included in the SI.

### Microbial Community Analysis

Twice a week, 5.4 mL of broth was collected for microbial community analysis. Centrifuged cell pellets free of supernatant were resuspended in 1 mL of 10 mM Tris-HCl buffer (pH 8.5) and centrifuged for 10 min at 20,000*g*. Supernatant was removed and the pellets were stored at −20 °C for microbial community analysis.

DNA was extracted using the NucleoSpin Soil Kit (Macherey-Nagel, Germany) following the manual’s instructions and DNA quantification was conducted with Qubit dsDNA BR Assay Kit (Invitrogen, Germany). Amplicons covering the V3–V4 region of the 16S rRNA gene were generated using the primer set 341f (5′-CCT ACG GGN GGC WGC AG-3′) and 785r (5′-GAC TAC HVG GGT ATC TAA TCC-3′).^43^ The library was prepared with MiSeq Reagent Kit v3 with 2 × 300 cycles and sequenced on the MiSeq platform (Illumina, San Diego, CA).

Amplicon sequence analysis was done according to Baleeiro et al.^15^ Briefly, Cutadapt^44^ was used to remove primer sequences, and DADA2 to infer amplicon sequence variants (ASVs).^45^ Phyloseq^46^ was used for diversity analysis in R Core.^47^ All samples were rarified to an equal sequencing depth of 6,730 counts. Spearman’s correlation analysis was done between abundance data of ASVs and the production rates of the different metabolites for the whole data set (*n* = 120). For correlation analysis, the value of 1 was added to the rarified sequence counts to remove zeros^48^ and adapt it to a centered log ratio transformation to account for microbial community data compositionality.^49^ Raw sequence data was deposited at the European Nucleotide Archive (ENA) under the study accession number PRJEB88737 (http://www.ebi.ac.uk/ena/data/view/PRJEB88737).

### Techno-Economic Analysis

Detailed assumptions adopted in the techno-economic analysis are presented in the SI.

## Results and Discussion

Reactor operation for this study was performed semicontinuously for 208 days. The experiment was divided into phases that compared H_2_:CO_2_ vs N_2_:CO_2_ and syngas vs N_2_:CO_2_, each at high and low OLR, as well as varying pH and temperature. Table S1 presents the conditions for each operation period as well as rationales behind each. The reactors were restarted once on day 104. Representations in metabolite concentrations, absolute metabolite quantities, average production rates, and gas partial pressures as well as a detailed description of reactor conditions are provided in the SI.

### Initial Effects of H_2_, C_2_H_4_, and CO

Figure 1 presents the electron balance for the period between 70 and 91 d. In this phase, the reactor operated under low OLR. C_2_H_4_ was added to the test reactor previously, starting on day 49 (Figures S1 and S3). Figure S1 shows a decrease in CH_4_ partial pressure coinciding with the addition of C_2_H_4_, which supports previous work describing the ability of ethylene—a common syngas impurity—to inhibit methanogenesis.^13^ As a result of the suppression of methanogens, bacterial H_2_ consumers were able to emerge, as shown by a net H_2_ consumption observed in the test reactor, compared to a net production of H_2_ and higher CH_4_ production in the control (Figure 1). Still, the amount of H_2_ consumed at this stage was not enough to impact chemical accumulation. CO started to be injected on day 79 and initially maintained an average partial pressure of 2.0 kPa in the test reactor (Figure S1). An incipient consumption of CO is visible starting around day 86, about 7 days after its first addition, which indicates a short acclimation period (Figure 1).

**Figure 1:**
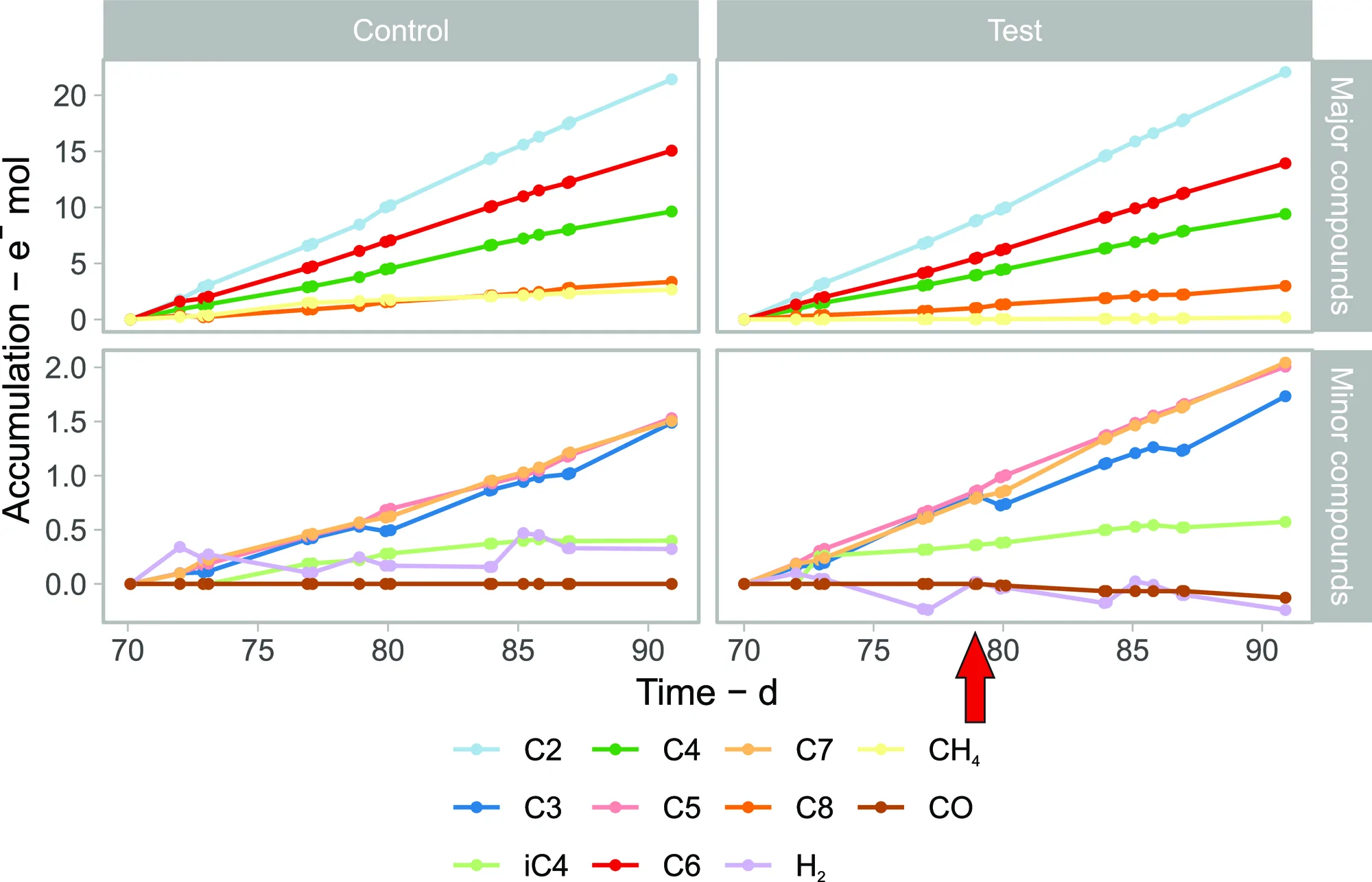
Accumulation of mol-electron equivalents over time for select gases and carboxylates between days 70 and 92. C_2_H_4_ was used as a methanogenesis inhibitor in the test reactor at this time; the red arrow on day 79 indicates the first CO addition to the test reactor. Gas balances prior to day 70 are not shown because tracer gas concentrations were not reliable enough to calculate accurate balances.

Despite the increased H_2_ partial pressures after C_2_H_4_ addition (average of 33.0 kPa for the whole period in comparison to 10.7 kPa in the control), only minor changes in the metabolite concentrations of the test reactor were observed: a slight increase of C2 and C4 concentrations and a decrease of C8 concentration (Figure S3). Regarding community composition during this period, under increased H_2_ partial pressure, genera such as *Prevotella, Syntrophococcus*, and *Dialister* had higher relative abundance compared to the control, while the relative abundance of *Lactobacillus* decreased under high H_2_ availability conditions (Figure S3). Outside of these differences, the two reactors showed similar community composition trends, which is reflected in their metabolite concentration similarities.

### Effects of Process Parameters on Metabolite Production

Among changes made throughout the experiment, OLR and the addition of CO had the strongest effect on MCC production, particularly on C2, C4, and C6 production. Figure 2 shows metabolite and gas production rates during periods of low OLR (days 104–146) and high OLR (days 180–208). At high OLR (7.2 g VS L^–1^ d^–1^), syngas did not appear to make a difference to metabolite concentration. Average C4 and C6 production were the highest under high OLR conditions (7.2 g VS L^–1^ d^–1^), with C4 and C6 produced at 4.30 and 4.31 mmol L^–1^ d^–1^ for C4 and 4.18 and 4.09 mmol L^–1^ d^–1^ for C6 in the control and test reactors, respectively (Figure 2A). Production rates of other metabolites, such as C2 and C3, were also higher at a high OLR. This could be attributed to the increased availability of EDs related to an increased substrate availability, rather than effects of syngas addition vs no addition. In contrast, at low OLR, specifically on days 104–146, there was a clear difference in MCC production between the control and test reactors. At low OLR, the average C4 and C6 production rates in the test reactor were 4.72 and 2.16 mmol L^–1^ d^–1^, respectively, representing a 74 and 34% increase compared to the control over a 42-day period (Figure 2B). The increased MCC production in the test reactor coincided with H_2_ and CO consumption (9.35 and 0.88 mmol L^–1^ day^–1^, respectively), suggesting that H_2_ and CO consumption was important for C4 or C6 production. Net H_2_ consumption did not occur in the control reactor. The high average partial pressures of H_2_ (51 kPa H_2_, test reactor with a low OLR), combined with the presence of CO, might also contribute to some degree to stronger H_2_ consumption rates. Interestingly, in an earlier phase (days 70–91, low OLR), neither the addition of CO nor the addition of H_2_ appeared to affect the accumulation of C4 and C6 when comparing 20 days before and after the addition of CO (Figure 1). Compared to the control, the test reactor had lower C2 production, suggesting either lower rates of acetogenesis or higher rates of conversion of C2 to longer-chain carboxylates, such as C4.

**Figure 2:**
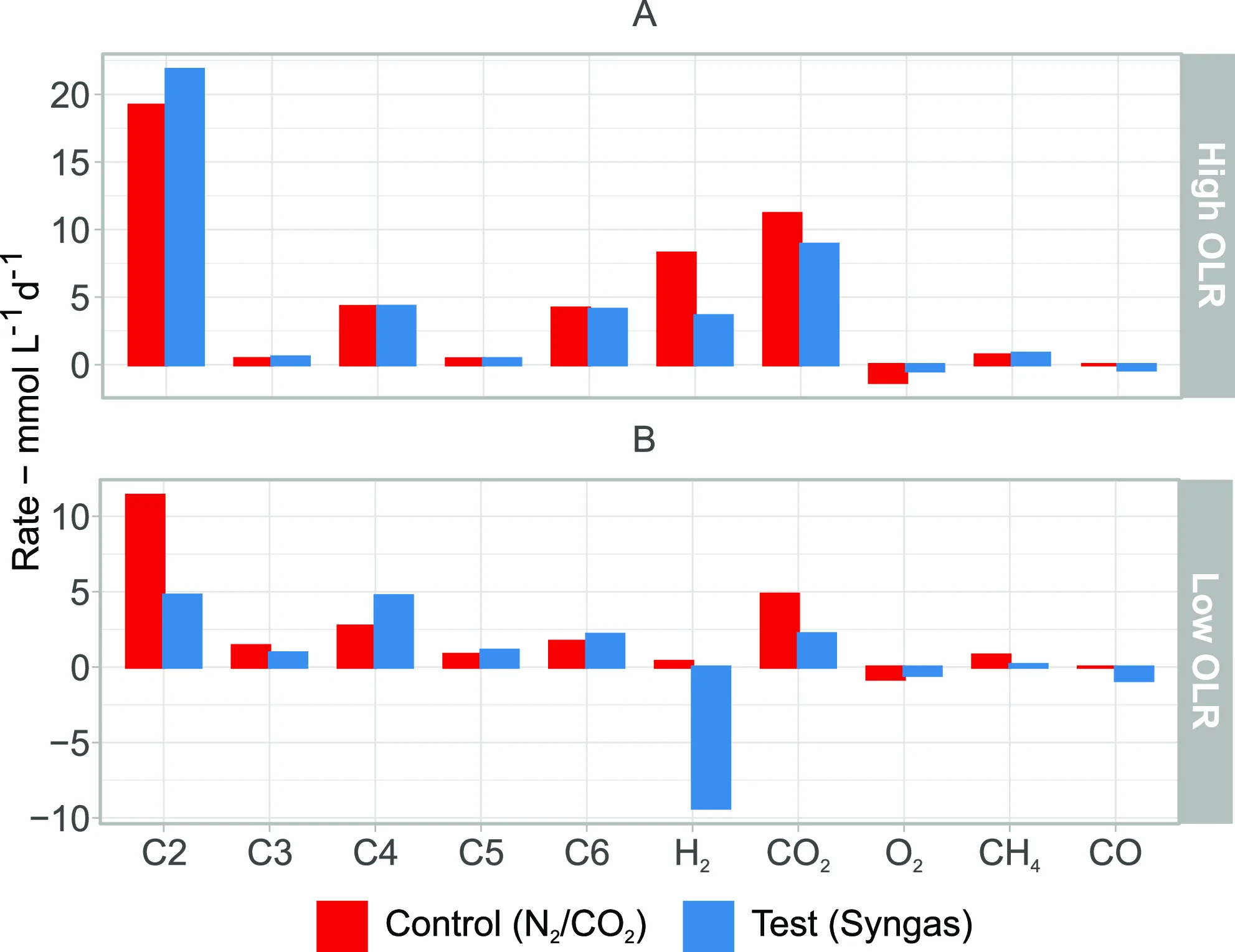
Average production and consumption rates for selected metabolites and gases between days 180–208, OLR = 7.2 g VS L^–1^ d^–1^ (A) and days 104–146, OLR= 3.6 g VS L^–1^ d^–1^ (B). Both reactors were operated with syngas during these periods. Negative rates denote consumption.

It should be noted that the community composition changed between these two phases, possibly as a result of a pH and temperature cycling experiment. Between days 146 and 180, pH and temperature were modulated to induce solventogenesis. To do this, pH and temperature were adjusted every 2.8 or 6.8 days between 32 °C/pH 5.5 and 28 °C/pH 4.8. C_2_H_4_, CO, and H_2_ were present in the test reactor at this time. PropOH and ButOH were used as solventogenesis indicators, but their concentrations remained minimal (<0.2 g L^–1^). Chemical concentrations were very similar in both the control and test reactors, pointing out that the reducing power of CO and H_2_ was not used to reduce carboxylates into alcohols to a relevant extent. It is possible that the low-pH phases were too short to allow for the microbial community to respond. Figure S6 presents the time profiles for chemical concentration, community composition, and pH variation for this experiment. An in-depth discussion about this experiment can be found in the SI.

### Community Members Involved in Syngas Consumption and CE

Following a reactor restart to readjust the reactor sparging system (see SI for more information), C4 and C6 were produced almost immediately in both the control and test reactors. At this point, both reactors were operating at low OLR, and the test reactor was operating with the addition of C_2_H_4_ and CO. The test reactor showed generally higher concentrations of C4 and C6 (averages of 3.3 and 2.1 g L^–1^, respectively) and lower C2 and C3 concentrations (3.1 and 0.59 g L^–1^, respectively) than the control (C4:2.0 g L^–1^, C6:1.7 g L^–1^, C2:6.1 g L^–1^, and C3:0.87 g L^–1^, Figure 3). *Megasphaera* was the key differentiating genus in the test reactor with syngas, which was not observed during the initial reactor stages. After an initial increase in both reactors, *Megasphaera* was outcompeted in the control reactor. In the test reactor, *Megasphaera* remained one of the three dominant genera, matching with increased C4 and C6 production (Figure 3).

**Figure 3:**
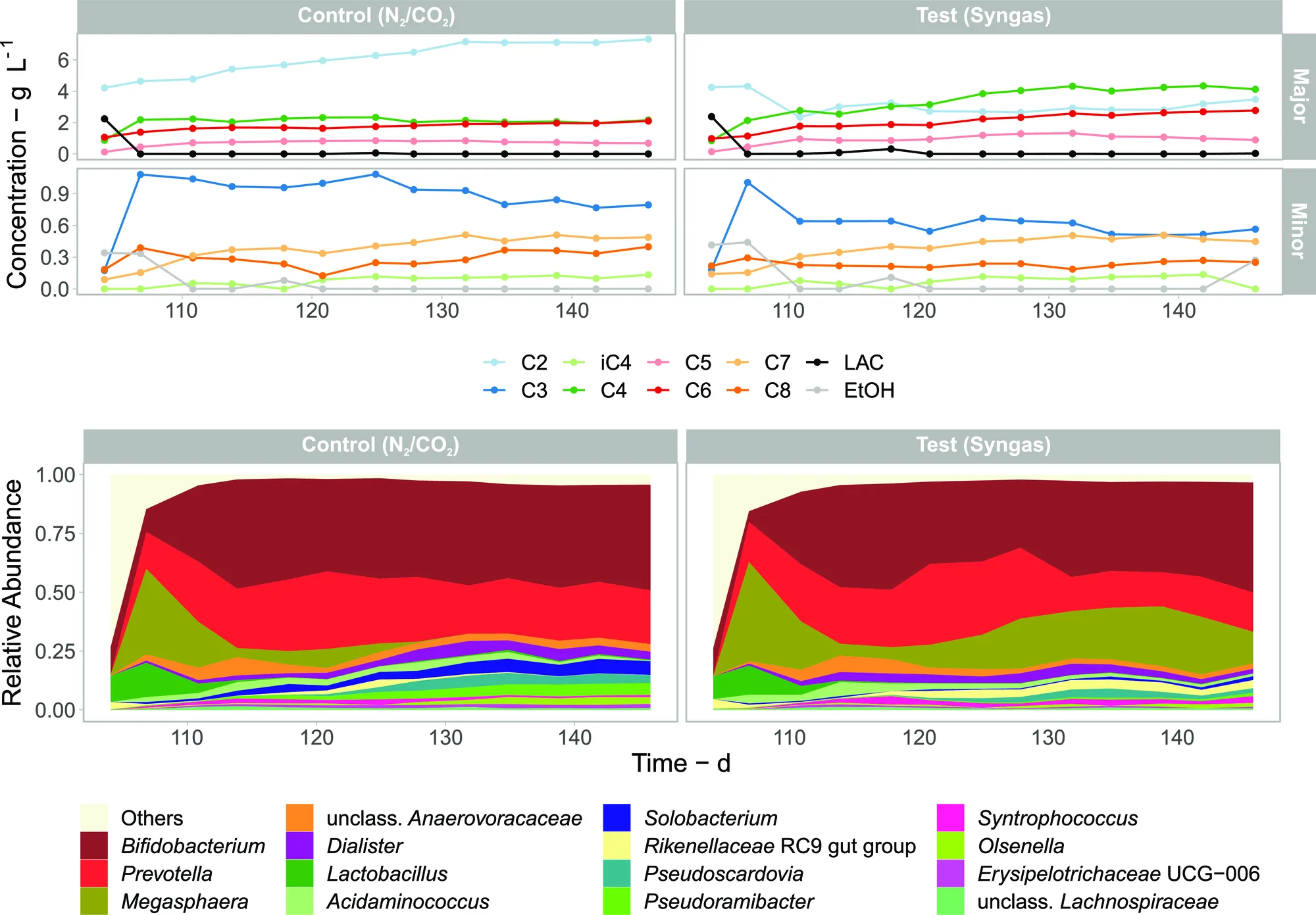
Chemical concentrations (top) and the top 15 most abundant genera present (bottom) over time when both reactors were operated at OLR = 3.6 g VS L^–1^ d^–1^, and the test reactor was operated with syngas.

Community composition was also affected by changing the OLR. High OLR (7.2 g VS L^–1^ d^–1^) and syngas were tested between days 180 and 208. The resulting community structure and chemical production are shown in Figure S5. Unlike low-OLR + syngas conditions, under high OLR, both reactors showed similar chemical and community profiles, with slightly higher C2 and C3 concentrations, lower relative abundances of *Olsenella* and *Caproiciproducens*, and a transient LAC accumulation in the test reactor. *Megasphaera* was not among the 15 most abundant genera from the beginning on day 181 until the end of this period on day 208, although it is not clear if its loss of relevance in the community was due to the high OLR or due to the pH cycling of the preceding experimental phase (Figure S6). A previous study^50^ showed *Megasphaera* being outcompeted after an OLR increase, although the genus was also reported to thrive in high LAC and low hydraulic retention time systems.^18,51^ Therefore, we would suspect that its absence under high OLR was simply because of a previous washout phase and the absence of a reinoculation before the start of the high OLR period (Table S1). Despite the washout of *Caproiciproducens* in the test reactor, the C6 concentration in both reactors was similar, suggesting that *Pseudoramibacter* was the main producer of longer-chain carboxylates under high OLR (Figure S6). *Pseudoramibacter* has been reported as a common chain-elongating genus in high-performing, low hydraulic retention time operation,^51^ in agreement with what we observed here. However, its potential could be better exploited in systems with lower C2 concentration.^52^

According to Spearman correlations (Figure 4), relative abundances of the ASVs *Megasphaera* (008 and 016), *Prevotella* (012 and 026), *Syntrophococcus* (014), and unclassified *Lachnospiraceae* (020) were significantly (*p* < 0.05) correlated to H_2_ consumption, whereas *Dialister* (015), *Megasphaera* (008 and 016), *Prevotella* (012), *Syntrophococcus* (014), and unclassified *Anaerovoracaceae* (022) correlated significantly with CO consumption. In terms of CO_2_ consumption, *Dialister* (015), *Megasphaera* (008), *Prevotella* (012 and 026), and *Syntrophococcus* (014) presented significant correlations (Figure 4). Among the ASVs correlated with gas consumption, *Megasphaera* (008 and 016) and *Prevotella* (012) were correlated to C3, C4, and C5 production, and *Dialister* (015) and unclassified *Anaerovoracaceae* (022) correlated to C7 production (Figure 4). Additionally, *Pseudoramibacter* (005), unclassified *Anaerovoracaceae* (003), *Olsenella* (019), and *Pseudoscardovia* (013) correlated to typical heterotrophic chain elongation: C6 or C8 fatty acid production without consumption of H_2_, CO, or CO_2_.

**Figure 4:**
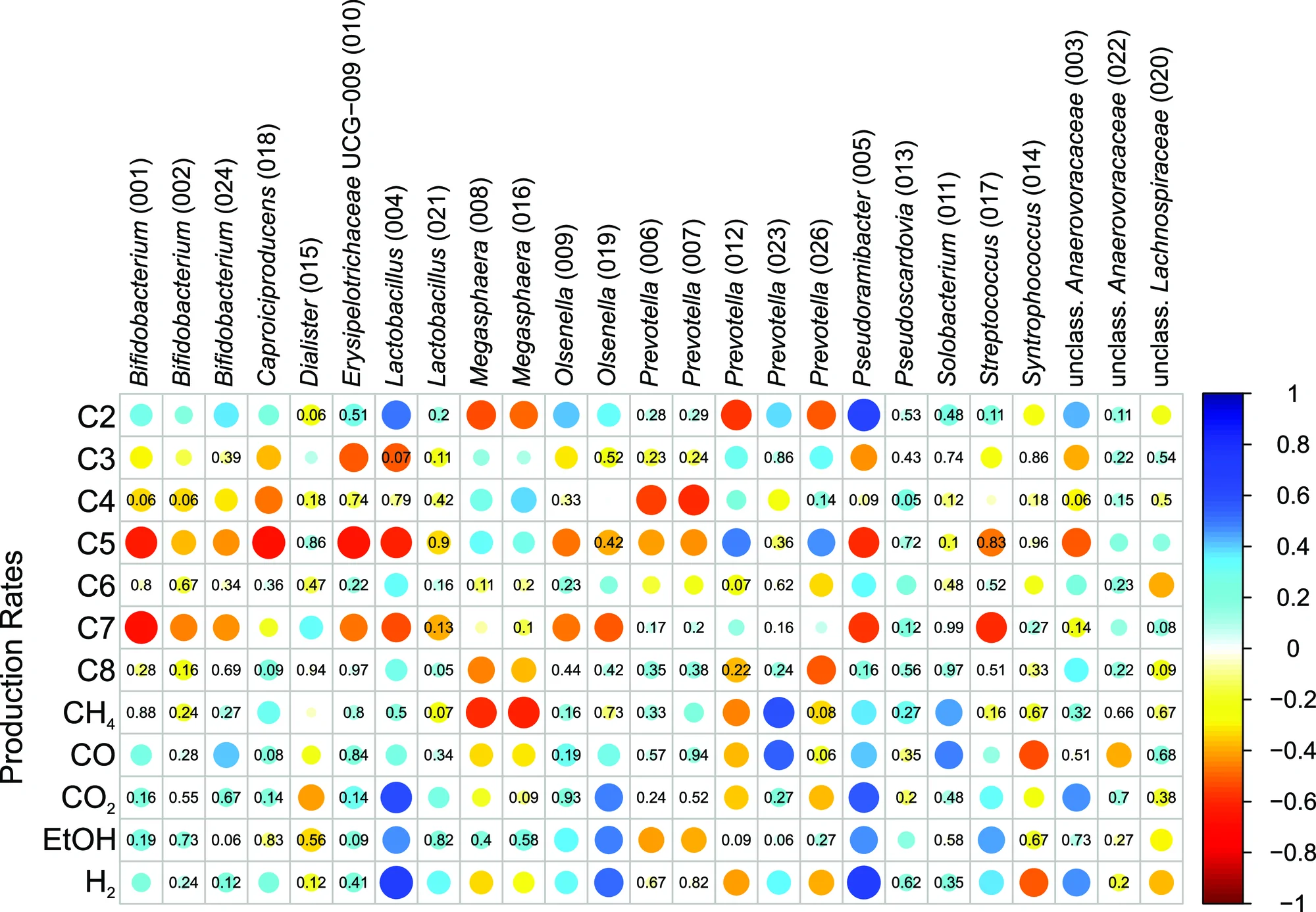
Spearman correlation between abundance of ASVs and production/consumption of selected metabolites across the entire experiment (*n* = 120). Blue shades indicate a positive correlation, showing that the ASV was abundant when the chemical production rate was high or when the consumption rate was low. Red shades indicate a negative correlation. Larger circles with darker shades indicate a stronger correlation between ASVs and metabolites. *p*-Values are only shown for the less significant correlations (*p* ≥ 0.05). ASV abundances were corrected for compositionality with a centered-log ratio transformation before calculating correlation coefficients.

Both *Megasphaera* and *Dialister* belong to the *Veillonellaceae*. While we previously discussed the overlooked potential of *Megasphaera* to consume H_2_ and grow mixotrophically,^15,17^ no carboxydotrophic members of this family are known. Still, many putative carbon monoxide dehydrogenase (CODH) genes have been found in genomes affiliated to the *Veillonellaceae*.^53^ The UniProt database^54^ returns 123 entries for CODHs and homologue proteins (e.g., hydroxylamine reductase) predicted or inferred from *Veillonellaceae* genomes (as of 04/28/2025 with the keyword “CODH”). These CODH genes are often accompanied by gene sets of the Wood-Ljungdahl pathway.^53^ Even though many of these gene sets lack the gene for formate dehydrogenase^53^ used to convert CO_2_ into formate, formate is a common intercellular metabolite, and we have previously shown its importance as an electron and carbon carrier between bacteria in mixotrophic fermentation of biomass and syngas.^16^ Therefore, we suspect that *Megasphaera* and *Dialister* compose the main mixotrophic group in our system. It is also possible—even though less likely—that both community members have different gas-consuming pathways despite their taxonomic relation. In open culture reactors without syngas addition, *Dialister* abundance has been shown to decrease when CO_2_ supply was removed,^55^ which could also point to a carbon fixation mechanism in it.

Considering ASVs that significantly correlated with gas consumption, there were two other candidate mixotrophic groups in our system: *Prevotella* (012 and 026) and the two *Lachnospiraceae* ASVs: unclassified genus (020) and *Syntrophococcus* (014) (Figure 4). Similar to the *Veillonellaceae*, many members of the *Lachnospiraceae* have recently been found to harbor genes for the Wood-Ljungdahl pathway.^53^ However, the *Lachnospiraceae* ASVs had very low relative abundances in this study, and while they correlated significantly with H_2_ and CO consumption, we could not associate them with any carboxylate product (Figure 4). For the *Prevotella* ASV that presented some correlation with H_2_ consumption, an indirect correlation might be the best explanation. Putative lactic acid producers and hydrolytic groups, including *Bifidobacterium*, *Lactobacillus*, *Pseudoscardovia*,^56^ *Olsenella*,^57^ and *Prevotella*, are likely crucial for supplying intermediates to other acidogenic bacteria and should be particularly prone to cross-correlation with diverse metabolic functions. We previously observed and discussed these possible cross-correlations between H_2_ and CO consumption and lactic acid bacteria.^16^ For *Prevotella*, a search for CODH in UniProt only returns genes for homologue enzymes (*hcp* or hydroxylamine reductases).

*Megasphaera* was favored when enriched with H_2_, CO, and educts from lignocellulosic biomass as EDs (Figure 3). The species description of *Megasphaera elsdenii* mentioned its capabilities of consuming H_2_ and producing a wide range of carboxylates from C3 to C6.^58^ To our knowledge, hints pointing to a stable mixotrophic role of this genus in an open culture have only been reported very recently by Arhin et al.^18^ and Henry et al.^25^ Also noteworthy is the presence of two ASVs of *Anaerovoracaceae*, which is a family recently shown to harbor lactate-based chain elongators,^59^ that only share the same phylum to *Megasphaera*. Here, one ASV (022) correlated to C4 and C7 production and CO consumption, while the other (003) correlated to C6, C8, and H_2_/CO_2_ production. The dominance of distinct chain elongator groups such as *Megasphaera* and *Anaerovoracaceae* in this exploratory system could point to a ubiquitous potential of enriching mixotrophic open cultures that are industrially relevant. If mixotrophic communities prove as easy to enrich as here, there is an untapped potential of using syngas in biomass fermentation for shaping microbial communities that valorize C1 compounds and H_2_ into economically attractive products.

### OLR as a Factor for Reduced Carbon Emissions

One interesting outcome of this experiment was a reduced level of CO_2_ emission from the test reactor under mixotrophic conditions. By summing the molar production rates of CH_4_, CO_2_, and CO in Figure 2, a value for molar carbon emissions can be obtained. Average C emission rates in C mmol L^–1^ d^–1^ calculated for all experimental periods are available in the spreadsheet file provided in the SI.

Under low OLR (3.6 g VS L^–1^ d^–1^), the test reactor emitted on average 73% less carbon (1.50 C mmol L^–1^ d^–1^ compared to 5.61 C mmol L^–1^ d^–1^ in the control). In parallel, it produced 74% more C4, 23% more C6, and 79% less CH_4_ than the control reactor (Figure 2B). Under high OLR (7.2 g VS L^–1^ d^–1^), the reduction of carbon emissions was 21% (9.37 C mmol L^–1^ d^–1^ compared to 11.9 C mmol L^–1^ d^–1^ in the control), presenting a trade-off between biomass utilization and carbon emissions.

Regarding MCC production, at low OLR, relatively low MCC concentrations (2.2 g C6 L^–1^) were sustained in comparison to 4.3 g C6 L^–1^ achieved at higher OLR. In the recent literature, more than 10 g C6 L^–1^ has been reported under optimal chain elongation conditions.^60^ Despite the benefit of higher MCC concentration associated with high OLR, the reduced carbon emissions at low OLR should not be overlooked. Steady-state low MCC concentrations are not an insurmountable obstacle, as several in-line extraction technologies exist that are currently being scaled up^61,62^ and could overcome this potential drawback. To further mitigate increased downstream processing burden associated with low OLR, or to address challenges associated with incorporating syngas under high OLR, alternate strategies for coupling syngas postfermentation could be employed, such as postfermentation syngas addition demonstrated by Núñez-Valenzuela et al.^63^

It is important to note that the maximum syngas consumption rate observed in this study (229 mL d^–1^ L^–1^ H_2_ and 21.7 mL d^–1^ L^–1^ CO over 42 d) was likely limited by gas–liquid mass transfer, despite very high mass transfer coefficients (*k*_L_ *a*) enabled by gas recirculation. Assuming partial pressures of 80 and 5 kPa and Henry coefficients of 0.78 and 0.97 mmol L^–1^ bar^–1^ for H_2_ and CO,^64^ respectively, we estimate the lower boundaries for *k*_L_ *a* values of 622 h^–1^ for H_2_ and 762 h^–1^ for CO for our system. These *k*_L_ *a* values would rank these stirred-tank reactors equipped with off-the-shelf aquarium spargers (Figure S4) in the same tier as high-performing microbubble, trickle bed, and hollow fiber membrane reactors.^65–67^ *k*_L_ *a* values are typically measured in relatively clean, homogeneous systems with growth media or cell broths. Therefore, it could be possible that the presence of stirred solid chunks from corn silage in the medium decreases bubble coalescence or increases gas hold-up or bubble retention time, causing the observed *k*_L_ *a* values. Testing this hypothesis was outside the scope of this study, and investigating these mechanisms and whether they can be exploited in similar fermentation systems is recommended.

Another reason why our system is estimated to be below its maximum capacity is the relatively low partial pressures of H_2_ and CO (Figure S1). The gas reservoirs used here were relatively small in comparison to the reactor volume (1.8:1 gas-to-reactor volume ratio), causing H_2_ and CO partial pressures to drop quickly in the case of consumption or dilution with biogas and inert gases. Higher gas-to-reactor volume ratios and more frequent refilling procedures will facilitate fermentation with a higher syngas consumption activity. Therefore, we assume that a fermentation with a net carbon fixation balance and a stronger effect of syngas on carboxylate production is technically feasible. Nevertheless, mixotrophic fermentation under CO partial pressures higher than those in this study needs to be studied further. In unacclimatized communities in batch systems, CO partial pressures as low as 5 kPa have been shown to inhibit CE activity,^16^ whereas other studies reported higher inhibiting thresholds above 25 kPa.^18,68^ Long-term community acclimatization through enrichment has been demonstrated with up to 100 kPa CO for producing ButOH and HexOH.^30^

### Turning Fermentation into an Industrial Carbon Sink

The ability of a mixotrophic fermentation process to simultaneously utilize syngas and reduce CO_2_ emissions compared to fermentation without syngas has significant implications for the potential of this technology to reduce environmental impact. One possibility is the creation of decentralized industrial carbon sinks by the low-tech conversion of anaerobic fermenters into mixotrophic gas fermenters.

In comparison to a conventional anaerobic bioreactor that only ferments biomass, mixotrophic operation adds three major layers of operational complexity: (1) H_2_ and CO have to be supplied or generated in situ; (2) continuous gas recirculation means that a blower or compressor needs to be employed; and (3) inert gases and impurities (e.g., N_2_, CH_4_, NH_3_, and H_2_S) need to be removed from the recirculating gas to avoid indefinite accumulation. Each of these points is associated with capital costs, operational costs, and environmental impacts. Here, we try to gauge the size of operational costs and emissions associated with points (1) and (2).

To calculate the costs associated with point (2), a range of the main factors for compressor electricity consumption (i.e., pressure increase and gas flow) has to be defined. In this study, a constant gas recirculation rate of 9.0 “reactor volumes” per hour (9.0 vvh, in h^–1^, equivalent to 0.15 vvm, in min^–1^) and an average pressure increase of 70 mbar were used. These values help set the upper and lower limits for gas flow rate and pressure, respectively. For reference, 9.0 vvh is 85,000-fold higher than the summed H_2_ and CO microbial consumption rates observed at low OLR (252 mL d^–1^ L^–1^ or 1.05 × 10^–4^ vvh). Figure 5 presents the expected operating costs and associated carbon emissions due to continuous gas recirculation rates from 1.05 × 10^–4^ to 9.0 vvh and compressor pressure increase from 70 to 2000 mbar. The expected operating costs of acquiring H_2_ and CO at spot market prices are shown in Figure 5A, while the range of avoided carbon emissions in kg CO_2 eq._ m^–3^ broth are shown in Figure 5B. The full set of assumptions adopted for these estimates is presented in the SI.

**Figure 5:**
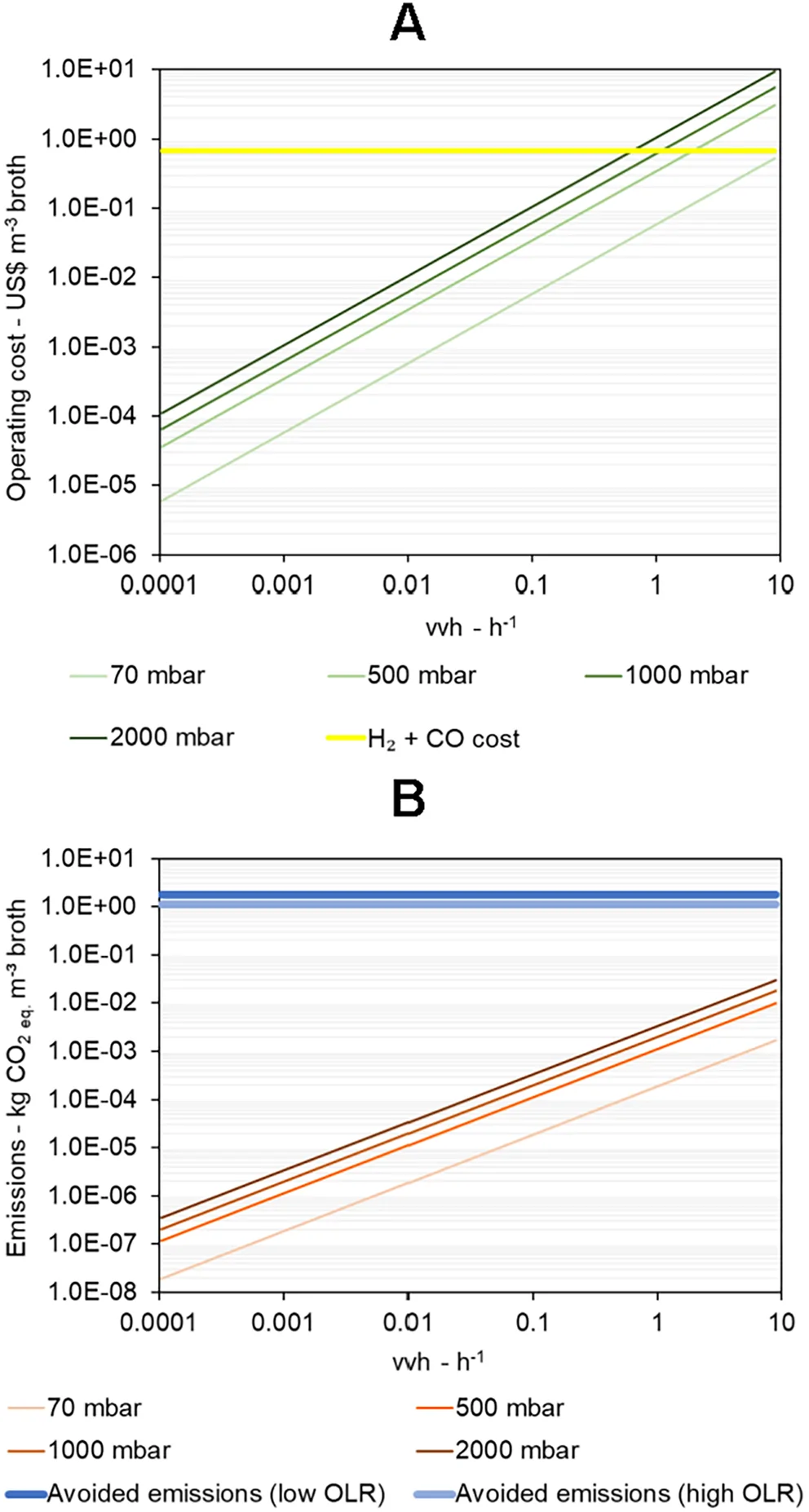
Operating costs of recirculating gas in USD m_broth_^–3^ (A) and carbon emissions in kg CO_2 eq._ m_broth_^–3^ (B) due to compressor electricity consumption depending on gas flow rate intensities in reactor volumes per hour (vvh) and pressurization needed.

According to Figure 5A, gas flow rates between 0.5 and 9 vvh are well within the operational range of industrial scale mixotrophic fermentation and would incur in operating costs that are in the same order of magnitude as the costs to supply blue or green H_2_ and CO, which are expected to be around 0.66 USD m_broth_^–3^. On the other hand, decreasing gas flow rates to below 0.1 vvh would result in gas recirculation costs that are orders of magnitude lower than the costs of supplying H_2_ and CO. However, we assume that using such low gas flow rates without decreasing microbial gas consumption rates would be technically challenging.

The impact of gas recirculation on carbon emissions should be framed differently than its impact on costs. Recirculating gas would be associated with carbon emissions no greater than 0.04 kg CO_2 eq._ m_broth_^–3^, which is well below the least of avoided C emissions that mixotrophy can provide (between 1.11 and 1.81 kg CO_2 eq._ m_broth_^–3^ according to our experiment) (Figure 5B). This is true even considering that our assumptions adopt a very conservative electricity emission intensity of 0.481 kg CO_2 eq_ kWh^–1^, which is about half the emissions of coal-generated electricity. Therefore, the avoided emissions due to microbial activity greatly outpace emissions due to electricity consumption regardless of gas recirculation flow rate and operating pressure.

Figure 6 presents possible operating costs for mixotrophic operation by combining gas circulation rates, compressor pressurization, and H_2_ and CO market prices to generate three scenarios with increasing techno-economic difficulty at the commercial scale. Assumptions for Figure 6 are described in the SI. Table S4 presents the extraction efficiencies for different carboxylates, based on Braune et al.,^69^ and the assumed carboxylate selling prices used to estimate broth value. Scenarios 1, 2, and 3 represent operating costs based on varying gas acquisition and electricity costs and are further described in the SI. These scenarios correspond to costs of 0.498, 1.26, and 2.11 USD m_broth_^–3^, respectively, and can be compared with possible broth values ranging from 7.0 to 14.4 USD m_broth_^–3^, depending on the operating conditions (Figure 6). In all scenarios, the value of the broth surpasses that of the operating costs. Operating the fermenter mixotrophically valorizes 1.40 USD m_broth_^–3^ by increasing C4 and MCC content and an extra 0.14 USD m_broth_^–3^, if carbon credits can be accounted for. At low OLR, mixotrophic fermentation would result in a total broth value of 8.54 USD m_broth_^–3^. Critically, a broth with lower MCC concentrations would incur higher product recovery costs. Moreover, the possibility of carbon credits depends on the system boundary used for carbon accounting.^70^ This proof of concept was done with corn silage, which is not a waste stream but an energy crop with the biggest relevance for the biogas industry in Germany.^71^ As mixotrophic fermentation technology matures, studies should perform life cycle assessments for each specific application case, considering the source of the organic feedstock, of the syngas components, application, and end-of-life of the MCC products, and integrated industrial plants such as steel works and biogas plants.

**Figure 6:**
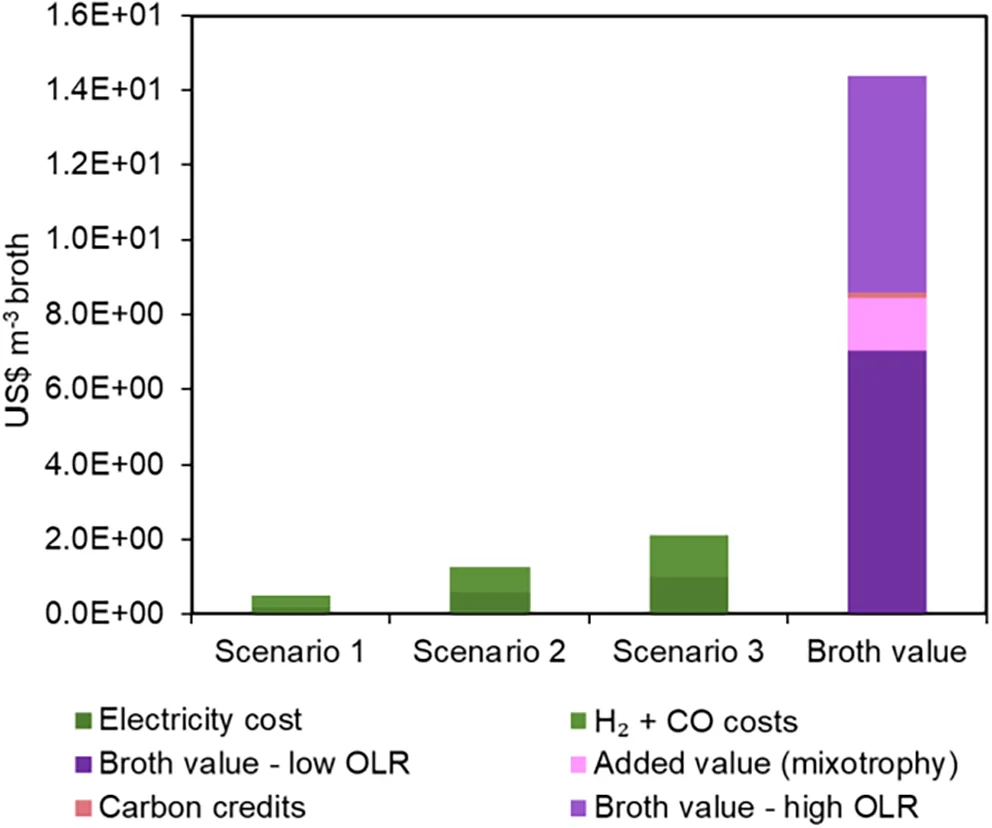
Comparison between the operating costs for supplying H_2_ and CO and continuously recirculating gas compared to the value of the carboxylates in the fermentation broth on a m^3^ of broth basis.

The presence of inert gases and impurities in the syngas will depend on its source and production technology.^72^ Nonwashable gaseous impurities with low boiling points, such as N_2_ and CH_4_, will likely impose particular constraints on the added complexity of syngas recirculation (point 3). These gases are not removed with state-of-the-art gas scrubbing, condensation, or adsorption technologies^72^ and might need more expensive membrane or cryogenic separation techniques in-line or at the syngas supply side. Alternatively, these inert impurities might impose a continuous purge on the recirculated syngas. Ultimately, this is an engineering problem, and its associated operating and investment costs should be estimated in the future.

It is important to note that the point of the feasibility analysis done here is to compare mixotrophic operation with a conventional carboxylate fermentation without syngas. In other words, all operating costs that incur in conventional carboxylate fermentation (e.g., feedstock procurement, chemicals, and downstream processing costs) were not included here. Despite this, this study shows that mixotrophic fermentation has the potential of not only being environmentally favorable—by turning carboxylate fermenters into carbon sinks—but also economically viable. Under the techno-economic scenarios 1 and 2 (i.e., with electricity plus gas supply costs not higher than 1.26 USD m_broth_^–3^) mixotrophic fermentation via gas recirculation is expected to be economically feasible. The assumptions underlying these scenarios are well within realistic boundaries since they assume blue or green H_2_ prices not higher than 3 USD kg^–1^, gas flow rates of up to 1.0 vvh, and compressor pressurizations of up to 1000 mbar (see SI for more details).

## Conclusions

Through the operation of a semicontinuous 10-L fermenter with long-term microbial community enrichment, utilizing real lignocellulosic substrate, and a complete syngas mixture (i.e., H_2_/CO_2_/CO) with one impurity (C_2_H_4_), this study demonstrates mixotrophic fermentation for the production of MCCs at its highest technology maturity to date. There are several possible benefits in coupling microbial consumption of organic and inorganic feedstocks, such as steering selectivity to MCCs or their alcohols, achieving carbon fixation, achieving a feasible power-to-chemicals process, or decreasing biomass dependency, to name a few examples. Each of these goals combined with the industrial context of application defines the best strategy or use case in which to couple syngas components and electricity. Here, we showed some promise in fulfilling some of these goals simultaneously (steering MCC selectivity and carbon fixation) by using syngas while generating potentially useful insights into systems that could apply similar strategies, such as bioelectrochemical systems. The carbon fixation findings in particular were likely limited by the size of gas reservoirs and could potentially be increased by increasing gas reservoir size or investigating the gas-transfer mechanisms within the presence of semisolid feedstock. Testing these hypotheses is recommended for future studies.

This study also adds indirect evidence to the growing body of the literature elucidating the structure of the microbial communities behind mixotrophic fermentation, particularly the connection between *Megasphaera* and its role under hydrogen-enriched mixotrophic conditions. These insights, however, are limited by the techniques used for microbial community analysis (i.e., 16S rRNA amplicon sequencing), the relatively high complexity of our system, and the lack of biological replicates. To explore further the microbial mechanisms of mixotrophic fermentation, future studies are recommended to use experimental setups with defined substrates, pure cultures or synthetic consortia, or a single-pass gas supply. In addition, more compact experiments with biological replicates and adoption of techniques such as isotope tracing and advanced omics (e.g., metagenomics, transcriptomics, and proteomics) would help track the electron flow pathways and give statistical significance to the findings.

We also recommend further research on mixotrophic fermentation that deals with its current technical shortcomings and unknowns. In particular, solutions that (1) decrease the gas recirculation rates without decreasing microbial gas consumption, (2) increase the concentration of extractable carboxylates in mixotrophic communities, (3) sustain microbial gas consumption under higher OLR, and (4) investigate the equipment and installations needed for commercial-scale gas recirculation are needed.

## Supplementary Material





## Notes

Raw experimental data and calculated average production rates (XLSX)
Additional figures, tables, and text describing reactor operation conditions, reactor configuration, chemical concentrations, analyzed microbial data, assumptions and parameters for techno-economic analysis, and calculations for economics and mass balances (PDF)

## Abbreviations

MCC: medium-chain carboxylate
CE: chain elongation
EA: electron-acceptor
ED: electron-donor
HPLC: high-performance liquid chromatography
VS: volatile solids
TS: total solids
GC: gas chromatography
OLR: organic loading rate
ASV: amplicon sequence variant
C1: formate
C2: acetate
EtOH: ethanol
LAC: lactate
C3: propionate
PropOH: 1-propanol
C4: *n-*butyrate
iC4: *i-*butyrate
ButOH: 1-butanol
C5: *n-*valerate
iC5: *i-*valerate
PentOH: 1-pentanol
C6: *n-*caproate
iC6: *i-*caproate
HexOH: 1-hexanol
C7: *n-*heptanoate
C8: *n-*caprylate
CODH: carbon monoxide dehydrogenase
